# The effectiveness of school-based skills-training programs promoting mental health in adolescents: a study protocol for a randomized controlled study

**DOI:** 10.1186/s12889-019-6999-3

**Published:** 2019-06-07

**Authors:** Amanda W. G. van Loon, Hanneke E. Creemers, Simone Vogelaar, Nadira Saab, Anne C. Miers, P. Michiel Westenberg, Jessica J. Asscher

**Affiliations:** 1 Child and Adolescent Studies, Utrecht University, Heidelberglaan 1, 3584 CS Utrecht, the Netherlands; 2 Forensic Child and Youth Care Sciences, University of Amsterdam, Nieuwe Achtergracht 127, 1018 WS Amsterdam, the Netherlands; 3 Developmental and Educational Psychology, Leiden University, Wassenaarseweg 52, 2333 AK Leiden, the Netherlands; 4 Graduate School of Teaching (ICLON), Leiden University, Kolffpad 1, 2333 BN Leiden, the Netherlands

**Keywords:** Intervention, Randomized controlled trial, Effectiveness, School-based skills-training programs, Social skills, Performance anxiety, Mental health, Stress

## Abstract

**Background:**

Adolescence is a period of elevated stress sensitivity, which places adolescents at increased risk of developing mental health problems such as burnout, depression, anxiety, and externalizing problems. Early intervention of psychological needs and low-threshold care addressing such needs may prevent this dysfunctional development. Schools may provide an important environment to identify and address psychological needs. The aim of this protocol is to describe the design of a study aiming to evaluate the effectiveness of low-threshold school-based skills-training programs promoting the mental health of adolescents and to examine moderators of the effectiveness.

**Methods:**

A Randomized Controlled Trial will be conducted to examine the effectiveness of two school-based skills-training programs aiming to promote mental health by improving either skills to deal with performance anxiety or social skills. A multi-informant (i.e., students, parents, and trainers) and multi-method (i.e., questionnaires and physiological measurements) approach will be used to assess program targets (skills to deal with performance anxiety or social skills), direct program outcomes (performance or social anxiety) and mental health outcomes (i.e., stress, internalizing and externalizing problems, self-esteem and well-being), as well as specific moderators (i.e., student, parent and program characteristics, social support, perfectionism, stressful life events, perceived parental pressure, positive parenting behavior, treatment alliance and program integrity).

**Discussion:**

The current study will provide information on the effectiveness of school-based skills-training programs. It is of crucial importance that the school environment can provide students with effective, low-threshold intervention programs to promote adolescents’ daily functioning and well-being and prevent the emergence of mental health problems that negatively affect school performance.

**Trial registration:**

Dutch Trial Register number NL7438. Registered 12 December 2018.

## Background

Adolescence is a phase of rapid growth and development in physical and psychological domains [1]. During adolescence many changes occur simultaneously, including puberty and the transition to high school. At the same time, adolescence is a period of increased stress sensitivity [2–4], which contributes to adolescents’ increased risk for mental health problems, such as burnout, depression, anxiety and externalizing problems [5–8] and which may negatively affect the well-being of adolescents [9–11] and later developmental outcomes [12]. Stress also has a negative effect on academic performance [13–15] and can result in school absenteeism or dropout [16]. Addressing psychological needs at an early stage, for instance to deal with stress and stress-inducing factors, is of crucial importance to prevent the development of mental health problems, school dropout and dysfunction later in life. An environment particularly suitable to help vulnerable adolescents is the school environment. Intervening in the school-context may be particularly beneficial to schools as well-being and mental health have been positively associated with academic functioning [17].

A promising avenue to reach adolescents with psychological needs is by focusing on stress. As experiencing stress is part of normative development during adolescence [4], interventions focusing on the reduction of stress may be experienced by adolescents as a low-threshold and appealing way to address their psychological needs. Stress has been defined as the condition or feeling that results when individuals perceive that the demands of a situation exceed the individual’s personal, psychological, or social resources [18]. During adolescence, performance pressure as well as social situations at school may trigger feelings of stress, also referred to as academic and social stress [19].

Academic stressors that are frequently reported by adolescents are related to tests, grades, homework, expectations about school, expectations about their career and future life plans [20]. Academic stress is related to performance anxiety, where individuals experience fear of failure, the fear to be unable to meet certain expectations of themselves or others, or test anxiety. Improving skills to deal with academic stress through intervention programs can reduce stress in the school environment [21, 22]. Social stressors originate from an individual’s social environment and are caused by factors that disrupt the relationship with others, such as social rejection, isolation, disagreements or bullying [23]. Dysfunctional social interactions can trigger stress [24], hence, improving social skills by teaching adolescents to better communicate with others, might reduce perceived social stress.

The ability to cope with stress is very important and requires cognitive and behavioral efforts to control or reduce stressful experiences [25]. One way to thus counter the negative effects of adolescents’ stress is addressing either academic or social stress by offering preventative skills-training programs that provide adolescents with tools to effectively cope with stress and regulate emotions [26]. In order to effectively help adolescents who perceive academic or social stress and to promote their mental health, this protocol describes a study to examine the effectiveness of two school-based skills-training programs targeting skills to deal with performance anxiety or social skills.

Several studies implemented skills-training programs targeting performance anxiety, mainly focusing on test anxiety. Recent studies in secondary school students reported decreased test anxiety compared to controls after interventions targeting a combination of coping skills, relaxation techniques and study skills [27–31]. A reduction was also found in physiological stress [27, 31], internalizing problems [28] and behavioral problems [27], as well as increased self-esteem [27].

Recent studies demonstrated increased positive social behavior and improved social skills in secondary school students after mindfulness or social and emotional skills based interventions compared to controls [32, 33]. A reduction was found for perceived stress [32], problem behaviors [33, 34] and internalizing problems [35], as well as increased self-esteem [33–35].

In sum, previous studies targeting skills to deal with performance anxiety and social skills in secondary school students demonstrated promising results, for academic and social stress as well as for mental health outcomes. However, previous research shows several limitations. First, only few studies performed a randomized controlled trial (RCT) [30, 35], which means that in non-randomized studies there might be confounding factors or bias. Second, only few studies used a targeted small-group intervention [28, 30, 35], which means that in studies with universal interventions there might be minimal or no effect for at-risk students because their greatest effect is on low-risk students, who are not the desired target [36]. Moreover, these studies had relatively small sample sizes (*N* = 74 and *N* = 56) [30, 35] which precludes clear conclusions about the effects, or targeted youth that experienced a traumatic event [28], precluding conclusions about non-clinical community samples. Third, only a few studies investigated mixed-samples of students from diverse ethnic backgrounds [30, 31, 33], which is important to be able to draw conclusions that generalize to the whole population [37]. Hence, there is still insufficient evidence for the effectiveness of skills-training programs, especially for students in mixed-ethnicity community samples. Additionally, little is known about potential moderators of the effectiveness of school-based skills training programs, likely due to power issues. Which students benefit from such training programs and which factors contribute to their effectiveness?

Moderators are student, parent and program characteristics that are likely to affect the effectiveness of the training programs. Student characteristics that may affect the effectiveness of the skills-training programs include demographic characteristics (i.e., age, gender, ethnicity, educational level and socioeconomic status (SES)) and social support, perfectionism, stressful life experiences, severity of problems, perceived parental pressure and basal stress levels.

For instance, high levels of social support have been positively associated with well-being and mental health in children and adolescents [38, 39]. Moreover, high levels of social support have been associated with a more beneficial psychological treatment outcome in clinical samples [40, 41]. High occurrence of stressful life events has been associated with stress-related psychopathology and negative mental health outcomes, such as anxiety, depression and risk behavior [42–44] and predicts adverse treatment outcome [45]. On the one hand, high social support and low occurrence of stressful life events are associated with more beneficial outcomes. On the other hand, several studies showed larger program effects for adolescents with high initial problem severity [46, 47]. Following this line of reasoning, it may also be possible that students with low social support or with high occurrence of stressful life events profit most from the skills-training programs, because the sessions provide them with tools that are not provided by their social network.

Perceived stress positively correlates with perfectionism [48] and perceived parental pressure [49], which have been associated with mental health problems such as anxiety and depression [50–52]. Previous studies showed that perfectionism and maternal rejection (i.e., less emotional warmth), the latter associated with parental pressure [52], are related to less beneficial treatment outcomes in children and adolescents with anxiety or depression [53–56]. It is therefore expected that students with higher levels of perfectionism or students who perceive more parental pressure may benefit less from the skills-training programs.

Parent characteristics that may affect the effectiveness of the skills-training program include demographic characteristics (i.e., educational level) and positive parenting behavior. Positive parenting behavior increases adolescents’ social competence reflecting positive functioning at school including peer competence and attachment to school [57], and improves the relationship between parent and adolescent [58]. Because of this warm relationship, parents may be more involved in the life of their child and students may feel more confident to talk about the program at home. It is therefore expected that the skills-training programs are more effective for students with parents that show more positive parenting behavior.

Additionally, program characteristics, i.e., treatment alliance, program integrity and trainer characteristics such as ethnicity, experience and perceived competence, may moderate the effectiveness of the skills-training programs. Treatment alliance is the perceived bond between the participant of the skills-training program and the group leader (i.e., trainer), which is demonstrated to be positively associated with treatment outcome in depressive patients [53] and in children and adolescents with anxiety disorders [59, 60]. Hence, it is expected that high participant-trainer alliance contributes to a more beneficial outcome. Program integrity refers to the extent to which a program is implemented as originally planned, and is reported by very few studies [61]. Finding non-significant effects may not be caused by an ineffective program, but because a program is not carried out as intended [62]. Therefore, it is important to examine program integrity in order to correctly draw conclusions on the effectiveness of the skills-training programs.

**Fig. 1:**
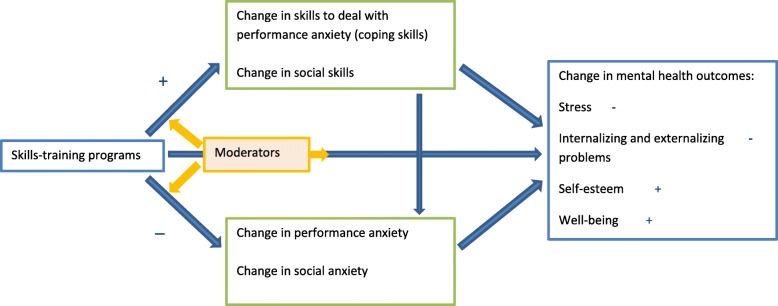
Conceptual model of the research design

In sum, the current protocol describes a study that will be conducted to examine the effectiveness of two types of targeted school-based skills-training programs addressing skills to deal with performance anxiety (program 1) and social skills (program 2). In order to overcome some of the limitations of previous studies, small-group skills-training programs are evaluated in a mixed-ethnicity community sample of students with different educational levels, where students self-select to one of the skill-training programs. For each program, a RCT is performed to examine if these low-threshold interventions reduce performance or social anxiety and promote students’ mental health. Both skills-training programs will be compared to a control waitlist condition. As illustrated in Fig. 1, it is expected that the performance anxiety skills-training program will improve skills to reduce performance anxiety (i.e., coping skills, including negative thought restructuring and managing emotions), resulting in a reduction in students’ performance anxiety (i.e., fear of failure and test anxiety) and in improved mental health (i.e., reduced stress and internalizing and externalizing problems, and increased well-being and self-esteem). Additionally, we expect that the performance anxiety skills-training program will directly reduce performance anxiety and improve mental health. It is expected that the social skills-training will improve students’ social skills and thereby reduce their social anxiety and improve their mental health (i.e., reduced stress and internalizing and externalizing problems, and increased well-being and self-esteem). In addition, we expect that the social skills training program will directly reduce social anxiety and improve mental health (see Fig. 1). The second aim is to investigate the moderators of the effectiveness of both skills-training programs on all outcomes. This is important because it is likely that the skills-training programs are not equally effective for all students. Student, parent and program characteristics will be examined as potential moderators. The final aim of this study is to evaluate if the school-based skills-training programs are experienced as sufficiently accessible, meaningful and helpful by the students, their parents and the trainers. Figure 1 shows a conceptual model of the research design.

## Methods/Design

### Design

**Fig. 2:**
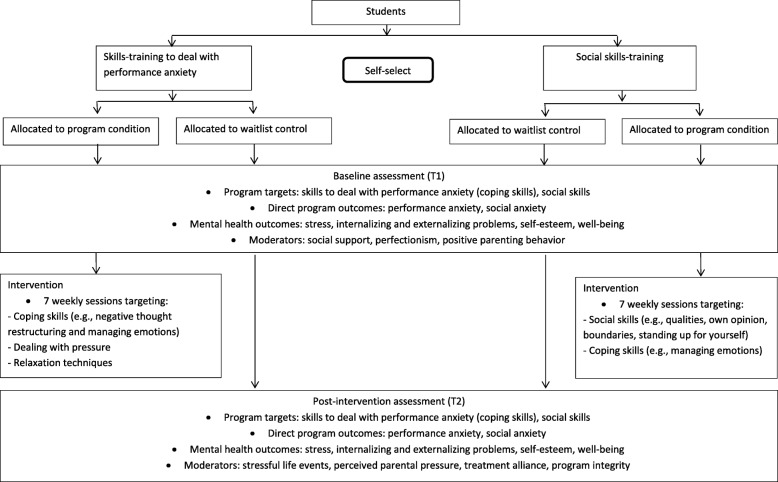
Flow chart of the research design

A RCT will be conducted for both school-based skills-training programs targeting: 1) skills to deal with performance anxiety and 2) social skills. For each skills-training program, students who have indicated interest in attending a training at their school, will be randomized by the first author (stratified for education level) into the experimental group in which the training starts immediately or into a waitlist control group that receives the training approximately eight weeks later (see Fig. 2), using computerized randomization in a 1:1 ratio. We will use a multi-method (questionnaires and physiological data) and a multi-informant (students, parents and trainers) design and will recruit a mixed-ethnicity sample of students from different educational levels.

### Data collection

Questionnaires assessing program targets (i.e., skills to deal with performance anxiety or social skills), specific goals of the skills-training programs (i.e., performance or social anxiety) and mental health (i.e., stress, internalizing and externalizing behavior, well-being and self-esteem) will be completed by students and parents prior to the start (T1) and after completion of the interventions in the experimental group (T2). Trainers complete questionnaires about program content (to assess program integrity) after each training session. Additionally, in a subsample of students (approximately *N* = 40 students per skills-training program), physiological parameters will be measured during a resting period to assess the basal stress levels of students (i.e., heart rate, heart rate variability and skin conductance measurements with wearables).

Student and parent characteristics (i.e., demographics including gender, age, ethnicity and education level, and expectations about the program) are assessed prior to the interventions, as well as trainer characteristics (i.e., demographics including gender, age, ethnicity, education level, and level of experience and perceived competence). Lastly, after completion of the intervention, students, parents and trainers will evaluate the skills-training program.

The design of this study has been approved by the Ethical Committee Psychology of Leiden University (CEP18–1105/419) and is registered in the Dutch Trial Register (number NL7438). To maintain participant confidentiality, all records that contain names or other personal identifiers will be stored separately from the collected data identified by code numbers.

### Study sample

For each school-based skills-training program we aim to include *N* = 130 students to ensure there is enough power for the analyses (*N* = 65 for both the experimental and waitlist control group). A total of *N* = 260 students will be included. This sample size is sufficient to investigate the effectiveness of the interventions and potential moderators, with a power of .80, an alpha of .05 and a medium effect size of .25 [63].

Participants are mixed-ethnicity students in the first, second, and third year of secondary schools (7th, 8th and 9th Grade students) of at least three schools in the Netherlands that offer education at various levels (from vocational education level to preparatory university education level). The students will be between 11 and 16 years old.

### Recruitment

#### Schools

So far, three Dutch urban secondary schools have been recruited. Possibly, additional schools will be recruited via the researchers’ contacts and networks. Schools that show interest will receive information about the study and will be asked to participate.

#### Participants

This study will be performed in the context of a Response to Intervention model (RTI) that aims to identify vulnerable students and provide them with appropriate interventions [64]. Before entering the current study, focusing on the effectiveness of school-based skills-training programs, classes of students receive educative information about stress and are asked about their own stress levels (i.e., Tier 1 of the RTI model: universal intervention targeting all students). After these three educative lessons, students will be asked to indicate if they would like to learn more about dealing with academic or social stress by following a skills-training program. Students will be asked to self-select to one of the school-based skills-training programs (i.e., skills to deal with performance anxiety or social skills), if needed assisted by parents or teachers (i.e., Tier 2 of the RTI model: targeted intervention directed at self-selected at-risk students). Students who self-select to a Tier 2 intervention are asked to participate in the current study.

The skills-training programs are offered by schools via their own care system or by external youth care organizations. The interventions are implemented at school by trained teachers or professionals. Students and parents receive written information about the different skills-training programs that are offered and receive an information letter about the corresponding research study. Trainers also receive an information letter about the study. Students and parents will be asked to provide active informed consent for the student’s participation in the study. If students or parents do not give consent to participate, the student will not receive the skills-training program offered by the study but will receive help via the schools’ own care system. Active informed consent will also be obtained from parents and trainers for their participation. After receiving consent, students will be randomized into the experimental condition or the waitlist condition. Figure 2 shows the flow chart of the design of the study.

### Interventions

Students in the experimental condition of each skills-training program will participate in seven 45-min small-group sessions during consecutive weeks. Weekly sessions will take place at the school of the students and the sessions will be delivered by an experienced trainer or trained teacher in small groups. The performance anxiety skills-training program consists of cognitive coping strategies (e.g., negative thought restructuring and managing emotions), relaxation techniques and dealing with pressure. The social skills training program consists of social skill building (e.g., identifying personal qualities, giving own opinions, setting boundaries, and standing up for yourself) and cognitive coping strategies (e.g., managing emotions).

### Waitlist-control group

The waitlist group will not receive any training during the implementation of the intervention in the experimental group and will only complete the pre-and post-intervention measurements. The waitlist group will receive the intervention immediately after the post-intervention measurements, approximately 8 weeks later than the experimental group.

### Instruments

Table 1 presents an overview of the measurements used at each assessment point for the skills-training programs.

**Table 1:** Overview of the variables’ instruments and sources

Outcome	Variable name	Instrument	Time of measurement	Variable type	Source
Program targets	Coping skills	CERQ-short	T1, T2	Outcome	Students
	Social skills	SIG-A	T1, T2	Outcome	Students, parents
Direct program outcomes	Performance anxiety	PFAI, TAI	T1, T2	Outcome	Students, parents
	Social anxiety	RCADS, subscale social phobia	T1, T2	Outcome	Students, parents
Mental health outcomes	Stress levels	CSQ-CA	T1, T2	Outcome	Students, parents
	Internalizing and externalizing behavior	Y-OQ	T1, T2	Outcome	Students
	Well-being	WHO-5	T1, T2	Outcome	Students
	Self-esteem	RSES	T1, T2	Outcome	Students
Physiological stress response	Stress	Heart rate, heart rate variability, skin conductance	T1, T2	Outcome & moderator	Students
	Current stress and mood	VAMS	T1, T2	Outcome & moderator	Students
Demographics	Demographics	Developed for this study	T1	Moderator	Students, parents, trainers
Student characteristics	Social support	SSL-I	T1	Moderator	Students
	Perfectionism	CAPS-14	T1	Moderator	Students
	Stressful life events	Negative life events inventory	T2	Moderator	Students
	Perceived parental pressure	MIPS parental pressure subscale	T2	Moderator	Students
Program characteristics	Level of experience and competence of trainers	Developed for this study	T1	Moderator	Trainers
	Program integrity	Developed for this study	T2	Moderator	Trainers
	Treatment alliance	TASC	T2	Moderator	Students
Parent characteristics	Positive parenting behavior	VSOG subscale positive parenting	T1	Moderator	Parents

### Outcome measures

Program targets, direct program outcomes and mental health outcomes will be assessed before the start of the skills-training program and immediately after the completion of the program.

### Program targets

*Skills to deal with performance anxiety* will be measured with the Dutch version of the Cognitive Emotional Regulation Questionnaire – short form (CERQ-short) [65], completed by students. This instrument is a 18-item self-report measuring cognitive related coping (e.g., “I think I can learn something from the situation” or “I keep thinking about how terrible it is what I have experienced”). It consists of nine subscales: Self-blame, acceptance, rumination, positive refocusing, refocus on planning, positive reappraisal, putting into perspective, catastrophizing and other-blame. The authors made a distinction between maladaptive coping (self-blame, other-blame, rumination and catastrophizing) and adaptive coping (acceptance, refocus on planning, positive refocusing, positive reappraisal and putting into perspective) [66]. The internal consistency of the subscales is between .68 and .81 [65].

*Social skills* will be measured with the Scale for Interpersonal Behavior of Adolescents (SIG-A) [67, 68], completed by students and parents. For parents, an adapted version will be used where “I” is replaced with “my child”. The self-report version for students consists of 47 situations that are evaluated on two dimensions (i.e., how much anxiety students experience during these situations and how often they experience these specific situations). In this study we only use the performance dimension (i.e., frequency) to assess social skills. For example, the item “Starting a conversation with someone you haven’t met before” is scored on a scale from “never” to “always” for the performance dimension. The instrument consists of four subscales that refer to specific social situations: 1) display negative feelings (14 items, e.g., “If someone interrupts you, saying you find that annoying”) 2) express personal limitations (13 items, e.g., “Asking for an explanation about something that you didn’t understood”), 3) initiate assertiveness (9 items, e.g., “Starting a conversation with someone you haven’t met before”) and 4) display positive feelings (8 items, e.g., “Agreeing when someone makes a compliment about your appearance”). This instrument has sufficient psychometric properties with a Cronbach’s alpha above .80 for all subscales [68].

### Direct program outcomes

*Performance anxiety* will be measured by two instruments measuring different domains, i.e., fear of failure and test anxiety, completed by students and parents. For parents, adapted versions are used where “I” is replaced by “my child”. The short form of the Performance Failure Appraisal Inventory (PFAI) [69] will be used, translated into Dutch. The PFAI is a 5-item self-report instrument measuring fear of failure (e.g., “When I am failing, I am afraid that I might not have enough talent”) with good reliability (internal consistency between .72 and .82) [69, 70]. The Dutch short version of the widely used Spielberger Test Anxiety Inventory (TAI) [71, 72] will be used to assess anxiety in school testing situations. This instrument consists of 20 items (e.g., “I feel confident and relaxed while taking tests”) and has demonstrated adequate internal consistency (between .92 and .96) [73].

*Social anxiety* will be measured with the social phobia scale of the Dutch version of the Revised Child Anxiety and Depression Scale (RCADS) [74], completed by students and parents. For parents, an adapted version will be used where “I” is replaced with “my child”. This instrument consists of 9 items (e.g., “I worry what other people think of me”) and has good internal consistency (between .78 and .81) [74, 75].

### Mental health outcomes

*Stress levels* of students will be measured with the Chronic Stress Questionnaire for Children and Adolescents (CSQ-CA) [5] and will be completed by students and parents (adapted version). The CSQ-CA is a 19-item self-report questionnaire (e.g., “I often get upset about things that are not important”) that demonstrated good psychometric properties with an internal consistency of .87 [5]. In addition, physiological measurements are performed to assess basal stress levels in a subsample of students. Heart rate, heart rate variability and skin conductance are measured via a wearable (Shimmer3 GSR+; [76]) during a 5-min resting period where students watch a relaxing aquatic video [77]. Prior to the physiological assessment, students will complete Visual Analogue Mood Scales (VAMS) [78] about their current mood and stress level.

*Internalizing and externalizing problem behavior* will be assessed with the Youth Outcome Questionnaire (Y-OQ-30.1) [79], translated into Dutch and completed by students. This instrument consists of 30 items (e.g., “My emotions are strong and change quickly”) and measures change in psychological symptoms and social functioning. It includes six subscales: somatic complaints (3 items), social isolation (2 items), aggression (3 items), conduct problems (6 items), hyperactivity/distractibility (3 items) and depression/anxiety (6 items). Internalizing problem behavior will be assessed with the subscale depression/anxiety and externalizing problem behavior with the subscales aggression and conduct problems. The internal consistency of the total scale is .92 and between .55 and .85 for the different subscales [79].

*Well-being* of students will be assessed with the Dutch version of the WHO-Five Well-Being Index [80], which consists of 5 items (e.g., “My daily life has been filled with things that interest me”). This instrument has good internal consistency for an adolescent sample (between .82 and .85) [81, 82].

*Self-esteem* is reported by students by completing the Dutch version of the Rosenberg Self-Esteem Scale (RSES) [83, 84]. The instrument consists of 10 items (e.g., “I take a positive attitude toward myself”) and has sufficient internal consistency (between .77 and .88) for high school students [83].

### Moderators

*Student, parent and trainer characteristics*, including age, gender, ethnicity and education level will be collected at baseline. Parents will also report family characteristics and information about work and education level (as indicator of SES), and trainers will report information about their level of experience, perceived competence and educational background. Finally, students and parents will report their expectations for the skills-training program (at baseline) and will evaluate the program (post-intervention measurement).

*Positive parenting behavior* will be measured with the Dutch Abbreviated Scale of Parenting Behavior (VSOG) [85], completed by parents. This instrument is a 25-item self-report for parents with five subscales. In this study only the subscale positive parenting behavior will be used, which consists of 8 items (e.g., “I make time for my child, when he/she wants to tell me something”). The subscale has a Cronbach’s alpha of .83 for mothers and .87 for fathers [85].

*Social support* will be measured with the Social Support List – Interactions (SSL-I) [86], adapted for use in adolescents. It measures the extent of received social support by social interactions in an individual’s social network. The instrument consists of 12 items (e.g., Does it ever happen to you that people: “are interested in you” or “ask you for help or advice”?) and three subscales (i.e., everyday social support, social support in problem situations and esteem support) and has acceptable internal consistency for all subscales (.70 or above) [86, 87].

*Perfectionism* will be measured with the Child and Adolescent Perfectionism Scale (CAPS-14) [88], translated into Dutch. This instrument is a 14-item self-report measuring perfectionism and consists of three subscales: self-oriented perfectionism-striving (3 items, e.g., “I try to be the best at everything I do”), socially prescribed perfectionism (7 items, e.g., “Other people always expect me to be perfect”) and self-oriented perfectionism-critical (4 items, e.g., “I get mad at myself when I make a mistake”). The internal consistency of the subscales is between .72 and .86 [88].

S*tressful life events* will be measured with the Negative Life Events Inventory [89, 90], translated into Dutch. This instrument is a 20-item checklist of negative life events (e.g., “Somebody in my family had a serious illness”). Students are asked to indicate whether an event had occurred during the previous year and includes events that occurred to family members and directly to themselves. The internal consistency is between .67 and .71 [89, 90]. Students will complete this instrument after completion of the skills-training program (post-intervention measurement).

*Perceived parental pressure* will be measured with the subscale parental pressure of the Multidimensional Inventory of Perfectionism in Sport (MIPS) [91], translated into Dutch. The perceived parental pressure subscale consists of 8 items (e.g., “My parents set extremely high standards for me”). The internal consistency is good (above .92) [91, 92]. Parental pressure has a high temporal stability [93] and students will therefore complete this instrument at post-measurement.

*Treatment alliance* will be measured with the Therapy Alliance Scale for Children (TASC) [94], translated into Dutch and altered for use in group skills-training programs. This is a 12-item instrument measuring the bond-aspect of alliance (6 items, e.g., “I like my trainer”) and the tasks of the program (6 items, e.g., “I work with my trainer on solving my problems”). This instrument has good internal consistency (above .70) [95, 96] and is completed by students after completion of the skills-training program (post-intervention measurement).

*Program integrity* will be measured with a questionnaire specifically developed for this study. After each session, trainers will be asked to evaluate the session by registering if they carried out the session as intended (e.g., is the content of the session sufficiently treated, are other components discussed, and what was their overall impression of the session).

### Statistical analyses

To examine the effectiveness of the skills-training programs, the effects of all outcome measures will be investigated by conducting analyses of covariance (ANCOVAs). The dependent variables are the outcome measures at post-test (program targets, direct program outcomes and mental health outcomes), the independent variables are the conditions and the covariates are the pre-test (baseline) measurements of the outcome measures. The effect of potential categorical and continuous moderators on the effectiveness of the skills-training programs on the specific goals of the programs and mental health of students will be examined by adding the moderators to the ANCOVAs. Data will be imported from an online server software (i.e., Qualtrics) and will be securely stored at the server of Utrecht University where data back-ups will be performed regularly.

## Discussion

This study protocol introduces the design of two RCTs investigating the effectiveness of school-based skills-training programs targeting skills to deal with performance anxiety or social skills to promote the mental health of adolescents. Further empirical tests of the utility of the RTI model will be published separately. Previous studies demonstrated reduced stress levels, reduced internalizing and externalizing behavior, increased self-esteem and improved well-being in adolescents receiving skills-training interventions. To overcome the limitations of previous studies, including study design (e.g., non-randomization, small sample sizes and the absence of moderator analyses) and use of mostly universal interventions, we will investigate small-group skills-training programs in a mixed-ethnicity community sample and use a RCT to exclude confounding (e.g., allocation and selection bias) as much as possible. We will use a multi-informant (i.e., students, parents and trainers) and multi-method (i.e., questionnaires and physiological measurements) approach. Furthermore, we will use a sufficiently large sample size to examine potential moderators of the effectiveness of the interventions (i.e., student, parent and program characteristics).

There are several challenges in this study, such as the recruitment of an ethnically diverse and representative sample of students and the prevention of dropout of students and parents. First, the self-selection of students to enroll in a skills-training program may constitute a challenge, as students may feel unable to make the correct decision or think they have problems in one domain, while these are caused by another underlying problem. We try to diminish this by involving parents and teachers to help students make the right choice. On the other hand, the self-selection of students may also be advantageous, because students are likely to be more motivated which may contribute to a more beneficial program outcome [97]. Second, it may be difficult to recruit sufficient numbers of mixed-ethnicity students and parents and maintain their involvement over the course of this study. Recruitment may be difficult because of language barriers and unfamiliarity and mistrust with research. It is therefore of great importance that the schools are actively involved in giving information to students and parents, because they rely and trust on their school. Since participation of students requires active informed consent from their parents, it is of utmost importance to reach all parents and provide them with clear information about study participation. In our efforts to reach students and their parents, we will use multiple sources (i.e., researchers and teachers or other school contacts) and multiple methods (i.e., information during classes, written information and phone calls) to provide them with information and to emphasize the relevance of the study. In order to make giving permission as easy as possible, participants can also give digital consent. Moreover, the questionnaires for students, parents and trainers are brief, clear and can be completed online, to avoid lost or non-returned questionnaires. These precautions are expected to increase the likelihood of participation and retention. In addition, with these precautions we aim to reduce the risk that socially disadvantaged groups, that are difficult to recruit and retain in health research [37], are underrepresented in our sample.

Third, a challenge in this study is to implement the skills-training programs at schools because of logistics (i.e., different locations and scheduling) and the participation and involvement of multiple parties (i.e., researchers, schools, students, parents, youth care organizations that provide the skills-training programs, and municipalities involved). With regard to the latter, multiple schools and multiple organizations offering skills-training programs are involved in this study. To strengthen the collaboration between the schools and the youth care organizations, we will organize regular meetings with all parties involved and aim to match the schools with suitable youth care organizations. With regard to logistics, it is very important that enrollment in the skills-training programs and arrangements in terms of schedules and locations will be timely communicated with the schools and youth care organizations. The researchers will try to overcome potential implementation issues by being present at the schools at least once a week, by giving weekly updates to the youth care organizations, and by being available for consultation at any time during the referral process and skills-training programs.

Fourth, the participating schools do not belong to the same municipality geographically, which complicates the financial conditions for the skills-training programs. To overcome financial issues, we will talk to different municipalities to obtain funding for the skills-training programs. Finally, it is important to be aware that in the Netherlands, school and youth care systems are completely separate, both organizationally as well as financially, which makes cooperation complicated. We try to overcome this challenge by giving regular updates and organize meetings with all parties involved, to promote a fruitful collaboration.

Overall, in order to overcome the practical challenges in this study, we aim to be as flexible as possible (e.g., being available for consultation at all times) towards the different parties involved. Of foremost importance is investing time in clear and timely communication with all parties in order to collaborate as effectively as possible. We try to involve the parties as much as possible by organizing regular meetings and actively involving them in the decisions in the research. Furthermore, we try to work according to a standardized research protocol as much as possible, to avoid miscommunications and ensure a positive and productive collaboration.

The current protocol describes a study that will investigate the effectiveness of school-based skills-training programs targeting skills to deal with performance anxiety or social skills promoting mental health in adolescents. It is of crucial importance that the school environment can provide students with interventions to help them cope with stress-inducing factors and to prevent the development of mental health problems, school dropout and dysfunction later in life.

## Abbreviations

ANCOVA: Analysis of Covariance.
CAPS: Child and Adolescent Perfectionism Scale
CERQ: Cognitive Emotion Regulation Questionnaire
CSQ-CA: Chronic Stress Questionnaire for Children and Adolescents
MIPS: Multidimensional Inventory of Perfectionism in Sports
PFAI: Performance Failure Appraisal Inventory
RCADS: Revised Child Anxiety and Depression Scale
RCT: Randomized Controlled Trial
RSES: Rosenberg’s Self Esteem Scale
RTI: Response to Intervention
SES: Socioeconomic Status
SIG-A: Scale for Interpersonal Behavior for Adolescents
SSL-I: Social Support Inventory-Interactions
TAI: Test Anxiety Inventory
TASC: Therapy Alliance Scale for Children
VAMS: Visual Analogue Mood Scale
VSOG: Dutch Abbreviated Scale of Parenting Behavior
Y-OQ: Youth Outcome Questionnaire
